# Racial/Ethnic Differences in Perceived Smoking Prevalence: Evidence from a National Survey of Teens

**DOI:** 10.3390/ijerph7124152

**Published:** 2010-12-02

**Authors:** Kevin C. Davis, James M. Nonnemaker, Hosanna A. Asfaw, Donna M. Vallone

**Affiliations:** 1 RTI International, 3040 Cornwallis Road, Research Triangle Park, NC 277009, USA; E-Mail: jnonnemaker@rti.org; 2 American Legacy Foundation, 1724 Massachusetts Avenue, NW, Washington, DC 20036, USA; E-Mails: hasfaw@legacyforhealth.org (H.A.A.); dvallone@legacyforhealth.org (D.M.V.)

**Keywords:** perceived smoking prevalence, youth smoking prevention, smoking perceptions, race/ethnicity

## Abstract

Prior studies show that perceived smoking prevalence is a significant predictor of smoking initiation. In this study, we examine racial/ethnic differences in perceived smoking prevalence and racial/ethnic differences in exposure to contextual factors associated with perceived smoking prevalence. We used cross-sectional time series data from the Legacy Media Tracking Surveys (LMTS), a national sample of 35,000 12- to 17-year-olds in the United States. Perceived smoking prevalence was the primary outcome variable, measured using an LMTS question: “Out of every 10 people your age, how many do you think smoke?” Multivariable models were estimated to assess the association between perceived smoking prevalence; race/ethnicity; and exposure to social contextual factors. Findings indicate that African American, Hispanic, and American Indian youth exhibit the highest rates of perceived smoking prevalence, while white and Asian youth exhibit the lowest. Minority youth are also disproportionately exposed to social contextual factors that are correlated with high perceived smoking prevalence. These findings suggest that disproportionate exposure to social contextual factors may partially explain why minority youth exhibit such high levels of perceived smoking prevalence.

## 1. Introduction

Adolescents have been shown to greatly overestimate actual smoking rates among their peers, regardless of their own smoking status [1–6]. School-level studies, for example, have found that perceived youth smoking prevalence rates are typically twice that of self-reported actual smoking rates and are generally much higher than national estimates of actual smoking among given age groups [3,7]. Perceived smoking prevalence also has been shown to be highly predictive of smoking initiation among youth in both longitudinal and cross-sectional studies (e.g., [8–10]). More recent studies further suggest that this relationship is consistent across racial/ethnic subgroups [11]. Associations between misperceptions and downstream behaviors have also been well-documented for alcohol, drug use, and other health and problem behaviors, including eating disorders [12–17].

Perceived smoking prevalence also represents a key indicator of youths’ social norms about tobacco use and has therefore been the basis of various norms-correction interventions implemented as part of broader tobacco control efforts in the US For example, the Vermont Tobacco Control Program implemented a mass media campaign targeting 10 to 13 year olds that utilized the message “8 out of 10” teens do not smoke [18]. Other tobacco control efforts including the American Legacy Foundation’s national “truth” campaign and interventions by state tobacco control programs in Florida and Minnesota have also targeted perceived smoking prevalence as a key outcome for change among youth.

Although overestimation of peer smoking prevalence is common among most youth, little is known about how this phenomenon differs across racial/ethnic groups. A limited amount of empirical evidence suggests that minority youth may hold greater misperceptions about smoking prevalence despite having generally lower actual rates of smoking compared to white youth. In a school-based study of Milwaukee youth, Leventhal, Glynn, and Fleming [3] found that nonwhite students held significantly more inaccurate perceptions about smoking, even after controlling for smoking status and other risk factors. This phenomenon may also contribute to demographic patterns in adult smoking as African American youth “catch up” to white youth with later initiation. Recent data on adult smoking prevalence in the US [19] show that white and African American adults have comparable rates of smoking. Given the predictive association between perceived smoking prevalence and smoking initiation, high misperception of smoking prevalence among minority youth nationally suggests this may be an important risk factor for later smoking initiation among minority youth.

The current evidence on perceived smoking prevalence among youth does not include national samples. The Leventhal *et al.* study [3], for example, was limited to a small sample of Milwaukee youth and is not nationally representative. In the current study, we examine racial/ethnic differences in perceived youth smoking prevalence more closely by using a national survey of youth to identify racial/ethnic groups that exhibit disproportionate levels of perceived smoking prevalence. We also investigate the role of social contextual factors that may lead to greater misperceptions among minority youth. Social contexts have been acknowledged as important determinants of behavior [20,21] and have been extensively investigated as a means to guide smoking prevention efforts [22]. In this paper, we demonstrate that many social contextual factors are also correlated with perceived smoking prevalence and that minority youth may be disproportionately exposed to many of these factors. Disproportionate exposure to the social contextual correlates of perceived smoking prevalence may therefore partly explain why minority youth are more likely to hold inaccurate perceptions about peer smoking prevalence.

Our study focuses on three primary research questions (RQs): (RQ1) How does perceived smoking prevalence and discrepancies between perceived and actual smoking prevalence differ by racial/ethnic groups among youth in the United States? (RQ2) Do minority youth have greater exposure to social contextual factors that are associated with high levels of perceived smoking prevalence? (RQ3) Does differential exposure to social contextual variables that are correlated with perceived smoking explain why minority youth exhibit the highest levels of perceived smoking prevalence? Findings related to each of these research questions are reported from a national survey of teens in the US and implications for future research and interventions are discussed.

## 2. Methods

This study used data from the Legacy Media Tracking Surveys (LMTS), a series of eight national telephone surveys of youth and young adults that were conducted between winter 1999 and fall 2003. The eight waves of LMTS had overall response rates of 52.5%, 52.3%, 60.4%, 46.7%, 51.7%, 53.1%, 42.5%, and 30.1%, respectively [23]. The LMTS was designed to track youth smoking behaviors, attitudes, and beliefs; awareness of pro- and anti-tobacco media messages; and a myriad of other background characteristics. The surveys contain a combined nationally representative sample of approximately 35,000 12- to 17-year-olds with increased representation of Hispanics (n = 6,293), African Americans (n = 5,174), Asians (n = 2,885), and a sample of American Indian youth (n = 674). Study analysis variables and our statistical analysis methods are described in detail below.

### 2.1. Perceived Smoking Prevalence

Perceived smoking prevalence was assessed with the LMTS item that asked “Out of every 10 people your age, how many do you think smoke?” This construct, and others like it, have been used in a number of other studies [4,24]. Responses ranged from 0 to 10, serving as the basis for the primary outcome variable considered in this study. Values of this variable were divided by 100 (*i.e.*, scaled) to represent an interpretable perceived peer smoking proportion. For example, answering “5” to this question is equivalent to estimating that 50% of one’s peers smoke. This aids in interpretation of model coefficients discussed later. As discussed previously, similar measures of perceived smoking prevalence have exhibited predictive validity in their association with smoking initiation [8–11].

Concurrent validity of this measure is also exhibited in the LMTS data. For instance, teens who indicate a high perceived smoking prevalence should have more friends in their immediate peer group who actually smoke. That is, smoking prevalence among the group of peers they reference when thinking about perceived prevalence should be higher. To establish this concurrence, we analyzed data on the LMTS questionnaire item “How many of your four closest friends smoke cigarettes?” We used the combined 8 waves of LMTS data to estimate perceived smoking prevalence by the number of 4 closest friends that smoke. As expected, there is a strong and statistically significant relationship between number of friends that smoke and perceived smoking prevalence. This association is also consistent by race/ethnicity. These patterns suggest that perceived smoking prevalence effectively distinguishes between youth who have a high number of close friends who smoke and those who do not (as we theorize it should).

Below, we describe independent variables that were included in our analyses based on their potential associations with perceived smoking prevalence and their availability in the LMTS data. For organizational purposes, these variables are grouped into four main categories (1) social environment factors; (2) exposure to pro- and anti-tobacco media messages; (3) school factors; and (4) community structural factors.

### 2.2. Social Environment Factors

The LMTS includes information on many social environment factors, such as living in a two-parent household, parental communication about tobacco, parental smoking, individual employment status, and religiosity. Living in a two-parent household was measured using a dichotomous variable to indicate whether the respondent lives with both parents. Parental communication about tobacco was measured as an indicator variable for whether either of the respondents’ parents discussed what they can and cannot do regarding tobacco during the past 6 months. To measure individual employment status, we created a simple dichotomous variable to indicate whether the respondent is currently employed either full time or part time at a job for pay. We also measured religiosity as an indicator for whether the respondent attends religious services often.

### 2.3. Exposure to Pro- and Anti-Tobacco Media Messages

Researchers have argued that overestimation of smoking prevalence is influenced by the increased salience given to the act of smoking in movies and television [25–28]. We measured exposure to tobacco imagery in the media with a dichotomous indicator variable for having often seen television shows or movies where someone was smoking during the previous week. Our analysis also included a continuous measure of daily hours of television viewing. We also assessed susceptibility to tobacco industry advertising and promotions by including a measure for whether or not the respondent owns any merchandise that is emblazoned with cigarette company names or logos. Finally, we assessed awareness of anti-tobacco media messages using measures of prompted, self-reported exposure to Philip Morris’ “Think. Don’t Smoke” (TDS) and the American Legacy Foundation’s “truth” campaigns, which aired during the study period [29,30]. Youth were asked “Have you seen or heard any anti-smoking advertising or campaigns with the following themes or slogans?” Campaign brand names including the “truth” and TDS campaigns were then read to each participant, and they were asked to indicate whether or not they recalled these campaigns. Youth who indicated “yes” to each campaign were defined as having prompted recall of the campaign. We created dichotomous indicator variables for recall of the “truth” and TDS campaigns separately.

### 2.4. School Factors

Almost all public and private schools in the United States implement a substance use prevention program funded by the Safe and Drug Free Schools and Communities Act [31]. These programs include tobacco use prevention education (TUPE) curricula, which emphasize the long- and short-term physical effects of smoking, the reasons why youth may smoke, and skills to refuse offers to smoke [32]. The LMTS measures self-reported exposure to each of these strategies. We also measured self-reported academic performance by creating a simple dichotomous variable indicating a perception that the student has done average or worse in school. While self-reported measures of academic performance are an imperfect proxy for actual grades [33], these measures have been shown to be as predictive of other outcomes as actual grades [34] and have been used in numerous studies of the relationships between academic achievement and smoking behaviors [35–41]. Although this construct may be limited by its self-reported nature, it serves as a reasonable proxy for factors related to academic performance, grades, or other aspects of the educational experience such as school connectedness that may be correlated with perceived smoking prevalence.

### 2.5. Community Structural Factors

Lastly, we measured several characteristics of each respondent’s zip code, including whether the respondent lives in the center city of a metropolitan statistical area (MSA), the percentage of the zip code’s population that has a 4-year college degree, and the median household income of the respondent’s zip code. These variables were drawn from Genesys Sampling Systems’ zip code–level data and were merged to the LMTS according to the zip code where each respondent resided.

### 2.6. Other Confounders

In addition to each of the contextual factors described above, our analyses included control variables for age, gender, and smoking status. Smoking status was measured as an indicator variable for whether the respondent had smoked on any of the 30 days prior to the survey. We control for smoking status because we want to determine the impact of specific social and contextual factors on perceived smoking prevalence independent of the contribution made by individual smoking status. This approach follows modeling strategies for perceived smoking prevalence that have been used in prior published research [11]. To account for the possibility that survey responses may be influenced by the presence of parents or others in the household, we included a variable for whether the telephone interviewer believed that someone else in the household was listening to the telephone interview. We also included a time trend variable equal to the number of months since the beginning of the LMTS to account for national trends in perceived smoking. Lastly, we included state-specific indicator variables to control for the potential association between perceived smoking and unmeasured state characteristics that are fixed over time.

### 2.7. Multivariable Analysis

Using the combined waves of the LMTS, we estimated a series of multivariable regression models that relate race/ethnicity and other individual factors, social environment influences, media influences, school factors, and community-level structural factors to perceived smoking prevalence. Our models were estimated using ordinary least squares (OLS) regressions. As noted earlier, the dependent variable (perceived smoking prevalence) was scaled such that regression coefficients on each of the race variables can be interpreted as percentage point differences relative to white youth (the reference group). Because the dependent variable is a discrete count, we estimated alternative sets of count-data models using both Poisson and negative binomial regressions. Results from these models were similar to the OLS results and did not indicate significant differences in the estimation strategy.

To examine how the contextual factors described above influence the relationship between race/ethnicity and perceived smoking prevalence, we estimated two models. The first model estimates the basic relationship between race/ethnicity and perceived smoking prevalence and includes control variables for only a minimal set of individual characteristics. In the second model, we introduce the complete set of control variables, including social environment factors, media influences, school influences, and community factors, to observe how the relationship between race/ethnicity and perceived smoking changes as contextual factors are accounted for. To better illustrate these changes, we then used the multivariable results to predict rates of perceived smoking by race/ethnicity from each of the two models. This provides a simple illustration of how racial/ethnic differences in perceived smoking prevalence change when differential exposure to all of the contextual factors is accounted for.

## 3. Results and Discussion

### 3.1. Results

#### 3.1.1. Descriptive Statistics

Descriptive statistics from the LMTS show that there are significant differences in perceived smoking prevalence by race/ethnicity among youth in the United States (Table 1). African American (46.6%), Hispanic (44.2%), and American Indian (48.4%) youth exhibited the highest rates of perceived smoking prevalence, whereas white (37.4%) and Asian (32.4%) youth reported comparatively lower rates of perceived smoking prevalence. Each white/Asian comparison to African American, Hispanic, and American Indian youth was statistically significant (p < 0.005).

Relative to white youth, African American youth were significantly more exposed to smoking imagery on television and in movies, were more likely to live in single-parent homes, watched more hours of television per day, had lower self-perceptions of academic performance, were more likely to live in areas with lower educational attainment, and were more exposed to parental communication about tobacco. Compared with white youth, Hispanic youth also watched significantly more hours of television per day, had lower self-perceptions of academic performance, were more likely to live in areas of low educational attainment, and were more exposed to parental communication about tobacco. American Indian youth also indicated higher levels of exposure to many of these factors. Specifically, compared with white youth, American Indian youth were more exposed to smoking imagery on television and in movies, were more likely to live in single-parent homes, watched more hours of television per day, had lower self-perceptions of academic performance, were more likely to live in communities with low educational attainment, were more likely to have parents that smoke, and were more exposed to parental communication about smoking.

Consistent with these findings, the LMTS data show that white and Asian youth reported significantly lower levels of exposure to each of the contextual factors described above. White and Asian youth were the least likely to live in single-parent households, were less likely to receive parental communication about tobacco, watched fewer hours of television per day, were less likely to recall images of smoking on television and in movies, had higher perceived academic performance, and were less likely to live in low-education communities compared with African American, Hispanic, and American Indian youth. Asian youth were significantly less likely than any other race to have parents that smoke.

#### 3.1.2. Contextual Influences on Racial/Ethnic Differences in Perceived Smoking

Table 2 summarizes two separate OLS regressions showing the association between perceived smoking prevalence and contextual factors that are measured in the LMTS. In both regressions, white youth are excluded as the reference group for each race/ethnicity coefficient. Each regression presents OLS coefficients for the association between each independent variable and perceived smoking prevalence. As described earlier, the outcome variable, perceived smoking prevalence, is scaled (divided by 100) to represent a perceived smoking proportion. Thus the OLS coefficients represent the percentage point difference between the independent variable characteristic and the reference group for dichotomized independent variables (e.g., race, gender, *etc.*) and the percentage point change given an increment change in continuous independent variables (e.g., age). The purpose of these models is to show how the estimated association between race/ethnicity and perceived smoking prevalence among youth changes when exposure to contextual factors are accounted for. Specification (a) adjusts for only a minimal set of individual characteristics that include age, gender, and current smoking status. Specification (b) introduces control variables for each of the contextual factors previously discussed.

Under specification (a), African American youth were estimated to report perceived smoking prevalence rates that were 8.4 percentage points higher than those reported by white youth, controlling for age, gender, and current smoking status (p < 0.001). Similarly, Hispanic and American Indian youth were estimated to report perceived prevalence rates that were 6.1 and 10.2 percentage points higher than those reported by white youth, respectively (p < 0.001). Conversely, Asian youth were estimated to report perceived prevalence rates that were 4.3 percentage points lower than those reported by white youth. With a minimal set of controls, these findings essentially reflect the descriptive statistics shown in Table 1.

When all available contextual factors are introduced into the model (Specification b), the estimated differences in perceived smoking prevalence by race/ethnicity are significantly reduced. The estimated difference, relative to white youth, was 4.7 percentage points for African American youth, 3.8 percentage points for Hispanic youth, and 6.6 percentage points for American Indian youth. The difference between Asian and white youth virtually disappears when all contextual factors are included and is not statistically significant. These results show that when the available contextual influences were accounted for, the total discrepancy in perceived smoking prevalence between white and other race/ethnic groups declined by 43.4% for African Americans, 37.9% for Hispanics, 34.9% for American Indians, and over 90% for Asian youth.

To better illustrate how racial/ethnic discrepancies in perceived smoking prevalence diminish when we account for differential exposure to contextual factors, we used the multivariable results from Table 2 to calculate mean predicted perceived smoking prevalence rates by race/ethnicity for each of the two models (Table 3). Specification (a) shows predicted perceived smoking prevalence adjusted only for age, gender, and smoking status, whereas specification (b) shows predicted perceived smoking prevalence adjusted for all available contextual factors. Although differences between white youth and African American, Hispanic, and American Indian youth remained statistically significant, the magnitude of racial/ethnic differences in perceived smoking prevalence declined dramatically when all available contextual factors were controlled for.

#### 3.1.3. Correlates of Perceived Smoking Prevalence

Our descriptive and multivariable results indicate that African American, Hispanic, and American Indian youth report significantly higher estimates of perceived youth smoking prevalence relative to white and Asian youth, but these differences significantly diminish when we adjust for other contextual factors.

Among the social environment influences included in our analyses, we found that parental smoking and parental communication about tobacco were positively associated with perceived youth smoking prevalence. We also found that living in a two-parent household and frequent attendance at religious services was associated with lower perceived smoking prevalence. The influence of parental communication is somewhat counterintuitive but may arise from the possibility that parent-child communication about smoking generates raises youths’ awareness of smoking in general and therefore may elevate their perceptions about actual smoking prevalence among their peer group. Thus, parent-child communication about smoking may lead to an inevitable correlation between this communication and perceived smoking prevalence. However, this is not to suggest that parents should not communicate with their children about tobacco use. While there may be a correlation with heightened perceived prevalence, there are other benefits to parent-child communication that would not be outweighed by these findings.

A number of media influences also were significantly associated with higher perceived smoking prevalence, including daily hours of television, frequent exposure to images of smoking on television and in movies, and ownership of pro-tobacco merchandise. Consistent with findings of a previous study [30], we also found that exposure to antismoking television ads from the “truth” campaign was associated with significantly lower perceived smoking.

We also found evidence of a significant association between perceived smoking prevalence and self perceptions of academic performance. Youth who believed that they performed at an average or below average level in school perceived a 5.83 percentage point higher smoking prevalence than youth who indicated they did better than average or much better than average in school (p < 0.001). This was the single most significant correlate of perceived smoking prevalence in terms of coefficient magnitude and likely reflects potential negative influences of poor school performance, low school connectedness, and potential poor quality of school instruction. We did not, however, find evidence of an association between our measures of exposure to school-level TUPE programs and perceived smoking prevalence (results not shown and are available upon request).

Finally, we found that community structural factors were, as a whole, less associated with perceived smoking prevalence. Of the structural factors we included in our model, we found that only average educational attainment within the respondent’s zip code was associated with perceived smoking prevalence. Specifically, we found that perceived smoking was lower in communities with higher rates of college graduation.

### 3.2. Discussion

With respect to RQ1, the results of this study show that there are significant racial/ethnic differences in perceived smoking prevalence on a national basis. Specifically, African American, Hispanic, and American Indian youth exhibit the highest rates of perceived smoking prevalence, whereas white and Asian youth exhibited the lowest. These differences still exist even after controlling for exposure to contextual factors that are correlated with perceived smoking prevalence. However, differences in perceived smoking prevalence by race/ethnicity decrease dramatically when these factors are accounted for in multivariable analysis.

Our second primary research question (RQ2) asks whether minority youth have greater exposure to specific social contextual factors that are associated with high levels of perceived smoking. Findings from our descriptive analyses confirmed that minority youth are significantly more exposed to a number of contextual factors that are positively associated with perceived smoking prevalence. These factors include living in a single-parent home, daily hours of television, exposure to smoking imagery in movies and television, poor self-perceived academic performance, and low community-level educational attainment, among others. Thus with respect to RQ3, increased exposure to contextual factors that are correlated with perceived smoking prevalence may partially explain why African American and Hispanic youth exhibit higher levels of perceived smoking prevalence compared with white and Asian youth, even though their actual smoking prevalence is significantly lower [42].

It should be noted that while the inclusion of these factors in our models significantly reduces the magnitude of the relationship between race/ethnicity and perceived smoking prevalence, a significant association remains. Thus it is possible, if not likely, that there are other factors unmeasured and unobserved in the available LMTS data that may account for the remaining relationships between race/ethnicity and perceived smoking prevalence. Further research with additional survey measures would be needed to explore this further.

Although disproportionate exposure to these social contextual factors may be a significant determinant of high perceived smoking prevalence among minority youth, many of these factors are “unchangeable” in the sense that they are inherent to youth’s social surroundings and are not conducive to interventions. Therefore, other intervention strategies may be needed to correct misperceptions about smoking prevalence among minority youth. For example, the use of “norms correction” media messages that convey accurate information about the true prevalence of smoking among youth is a potentially useful tool for countering misperceptions that most youth smoke. As described earlier, norms correction approaches have been used as part of several broad tobacco control efforts in the US For example, a recent study [30] showed that exposure to the national “truth” youth smoking prevention media campaign was associated with reduced perceived smoking prevalence among teens nationally and highlighted perceived smoking prevalence as a mediating factor through which media campaigns may affect smoking behaviors.

Our study also reveals new evidence on the problem of high perceived smoking prevalence among American Indian youth in the United States. Current cigarette use among high school students in schools funded by the National Bureau of Indian Affairs was 56.5% in 2001, roughly double the prevalence among all US high school students during the same time frame [43]. Other national studies, such as the National Survey on Drug Use and Health, have also shown that American Indian youth have the highest cigarette smoking prevalence among youth [44]. Given these data, in combination with previous empirical evidence of a predictive relationship between perceived smoking prevalence and smoking initiation, our findings that American Indian youth also exhibit the highest levels of perceived smoking prevalence are not surprising.

This study also highlights the debate over whether universal or specialized approaches to tobacco prevention are more appropriate for minority youth. Prior research has suggested that there are more commonalities than differences in the risk and protective factors associated with smoking among white, African American, and Hispanic youth [24,45–48]. These studies have also suggested that given these commonalities, universal intervention efforts may be more appropriate for youth of all races/ethnicities. Although there may be commonalities in the correlates of actual smoking behavior, theory-driven prevention programs typically seek to first influence the cognitive precursors of smoking, which can have their own risk factors that may differ significantly by race/ethnicity (as shown in the current study). Thus, when developing programs aimed at intervening on the precursors of smoking, it may be sensible to consider specialized approaches, such as media messages aimed at denormalizing tobacco, that are tailored to specific racial/ethnic vulnerabilities.

A few limitations to our study should be noted. First, declining response rates during the study period are a potential concern. Although declining response rates are a well-documented trend in telephone data collection, we do not believe our results have been biased because of this. With the exception of the last two waves of the LMTS, each survey achieved nearly a 50% response rate. In addition, the unweighted sample characteristics were virtually identical across all waves of data, suggesting there were no significant changes in sample composition over time. Furthermore, our main findings do not change significantly when we re-estimate our models excluding the last two waves of data with lower response rates.

Another potential limitation of this study is that due to the telephone survey mode, the rates of actual smoking prevalence shown in Table 1 are likely underreported. By extension, the gap between perceived and actual smoking prevalence may be overstated. Underreporting of risky behaviors like smoking prevalence in telephone surveys is well-documented in the survey research literature. This phenomenon is mainly the result of social desirability biases that exist when youth talk to a human interviewer. The impact of the telephone mode is also compounded by the possibility that a parent or other person in the household could listen to the interview. This is why we control for a measure of the interviewer’s assessment of the possibility that others are listening to the interview. However, while both of these factors contribute to general underreporting of smoking in telephone surveys, there is no evidence that patterns of underreporting differ by race in the LMTS data.

To further assess the possibility of racial/ethnic differences in behavioral underreports of smoking in the LMTS, we compared measures of current smoking in the LMTS to self-reported current smoking from the 2005 National Youth Tobacco Survey (an in-school self-administered survey of youth). In both surveys, current smoking is measured as having smoked cigarettes on at least 1 of the 30 days preceding the survey. Underreporting was measured as the percentage difference between the NYTS and LMTS estimates of current smoking. We found that white youth underreported smoking in the LMTS by an average of 59.4% compared to 52.0% among African Americans and 56.4% among Hispanic youth. None of these differences were statistically significantly, suggesting there are not dramatic differences in underreporting of smoking behavior by race.

Our findings pertaining to American Indian youth may also be limited by the LMTS sample design. The LMTS contains a national sample of youth who were interviewed *via* telephone. Thus, it is possible that American Indian youth who participated in these surveys may not largely reside in tribal regions, which are traditionally more difficult to reach *via* telephone survey methods, and therefore may not be representative of the American Indian youth population as a whole in the United States. If so, the reference groups upon which American Indian youth in the LMTS sample base their perceptions about perceived smoking prevalence may be different. As such, it is difficult to generalize our reported findings on perceived smoking prevalence among American Indian youth.

A final limitation of our study is that we only elucidate the external impetus for why perceived smoking might differ so significantly by race/ethnicity. Our data do not take into account racial/ethnic variation in adolescents’ own attitudes about smoking, nor do they take into account the value that adolescents place on others’ opinions about smoking. Prior research has suggested that parental opinions about youth smoking are important determinants of smoking behavior, regardless of parental smoking status [24]. To the extent that these factors vary by race, our analysis may present a somewhat incomplete picture of the sources of racial variation in perceived youth smoking prevalence.

## 4. Conclusions

This study presents new national data showing that minority youth are significantly more prone to high levels of misperception about youth smoking prevalence, a known predictor of smoking initiation. Much of this pattern can be explained by greater exposure to specific risk factors that are correlated with perceived smoking prevalence. Because many of these social contextual factors are inherent to youth’s social surroundings, tailored intervention approaches are needed to counteract the influence of these factors on perceived smoking prevalence. For example, interventions that feature norms correction approaches may be particularly effective in moderating the effects of misperceptions about smoking prevalence among minority youth.

## Figures and Tables

**Table 1:** Descriptive statistics showing exposure to contextual factors.

Contextual Factors	Race/Ethnicity				
	White	African American	Hispanic	Asian	American Indian
*Perceived and Actual Smoking*					
Perceived youth prevalence	37.4% [36.8–38.1]	46.6% [45.1–48.2]	44.2% [42.8–45.5]	32.4% [30.2–34.6]	48.4% [44.2–52.6]
Smoked in past 30 days	10.5% [9.6–11.4]	6.2% [4.6–7.7]	9.6% [8.0–11.2]	5.4% [3.2–7.5]	15.8% [10.2–21.3]
*Social Environment*					
Lives with both parents	77.4% [76.2–78.6]	48.4% [45.6–51.2]	74.0% [71.9–76.2]	86.2% [82.4–90.0]	57.7% [50.4–65.0]
Parent discussed tobacco in past 6 month	70.9% [69.7–72.1]	79.7% [77.4–81.9]	76.6% [74.4–78.9]	56.8% [52.3–61.3]	84.6% [79.8–89.4]
Either parent smokes	24.9% [23.7–26.1]	23.5% [21.0–26.0]	21.2% [19.2–23.2]	16.8% [13.1–20.5]	32.8% [26.0–39.5]
Attends religious services often	46.9% [45.5–48.3]	54.3% [51.5–57.1]	42.2% [39.8–44.7]	43.7% [39.0–48.3]	40.4% [33.2–47.6]
Currently employed full or part time	27.3% [26.1–28.6]	18.0% [15.8–20.2]	17.6% [15.7–19.5]	16.6% [13.4–19.8]	26.9% [19.9–34.0]
*Exposure to Pro- and Anti-tobacco Messages*					
Daily hours of television	3.03 [2.95–3.10]	4.30 [4.09–4.51]	3.50 [3.37–3.63]	2.90 [2.67–3.13]	3.56 [3.06–4.05]
Seen television/movie smoking often past week	52.2% [50.8–53.6]	59.7% [56.9–62.5]	52.2% [49.7–54.7]	45.7% [41.0–50.3]	63.2% [56.2–70.2]
Would use/wear pro-tobacco gear	16.7% [15.6–17.8]	13.7% [11.7–15.6]	17.4% [15.5–19.3]	12.1% [8.5–15.8]	18.5% [13.4–23.6]
Owns pro-tobacco gear	6.3% [5.6–7.0]	6.1% [4.8–7.4]	6.8% [5.5–8.1]	5.2% [2.3–8.0]	6.8% [3.9–9.7]
Has seen “Think. don’t Smoke” ads	71.8% [70.5–73.1]	60.2% [57.2–63.2]	68.3% [65.8–70.8]	68.7% [63.9–73.5]	67.7% [60.1–75.4]
Has seen truth ads	71.5% [70.1–72.8]	70.8% [68.0–73.6]	70.8% [68.3–73.4]	73.2% [68.5–77.9]	65.0% [56.9–73.2]
*School Factors*					
Exposure to tobacco use prevention education	76.8% [75.6–78.0]	79.7% [77.4–82.0]	78.5% [76.3–80.6]	80.0% [76.3–83.7]	78.0% [71.7–84.3]
Perceives school performance to be average or below average	39.1% [37.6–40.5]	46.0% [42.9–49.1]	45.8% [43.0–48.6]	27.5% [22.9–32.1]	47.7% [39.3–56.1]
*Structural Factors*					
Lives within center city of MSA	27.5% [26.3–28.7]	55.3% [52.5–58.1]	43.6% [41.1–46.1]	28.9% [25.1–32.6]	27.6% [20.8–34.3]
Percentage of zip code with college degree	20.0% [19.7–20.3]	16.5% [16.0–17.0]	17.2% [16.7–17.7]	26.6% [25.3–27.8]	16.2% [15.2–17.2]
Median household income in zip code (in thousands)	48.4 [47.9–48.9]	41.2 [40.4–42.0]	45.4 [44.6–46.3]	62.1 [59.8–64.5]	42.1 [40.3–43.8]

**Table 2:** OLS regression models showing the association between perceived smoking and contextual factors.

Explanatory variables	Specification	
	(a)	(b)
*Individual Characteristics*		
African American	8.36**	4.73**
Hispanic	6.08**	3.77**
Asian	−4.26**	0.31
American Indian	10.19**	6.63**
Other/unspecified race	2.20	2.54
Age	2.98**	3.38**
Male	−4.40**	−4.87**
Current smoker	16.39**	11.33**
*Social Environment*		
Lives with both parents	….	−3.11**
Parent discussed tobacco in past 6 months	….	2.85**
Either parent smokes	….	3.25**
Attends religious services often	….	−1.51**
Currently employed full or part time	….	1.15
*Exposure to Pro- and Anti-tobacco Messages*		
Daily hours of television	….	0.32**
Seen television/movie smoking often past week	….	5.03**
Owns pro-tobacco gear	….	5.93**
Has seen “Think. Don’t Smoke” ads	….	0.01
Has seen truth ads	….	−2.61**
*School Factors*		
Exposure to tobacco use prevention education	….	−0.15
School aptitude average or below average	….	5.83**
*Structural Factors*		
Lives within center city of MSA	….	0.48
Percentage of zip code with college degree	….	−1.74**
Median household income in zip code	….	−0.34

Note: All models include individual state indicator variables.

* Significant at *p* < 0.05.

** Significant at *p* < 0.01.

**Table 3:** Predicted perceived smoking prevalence by race adjusted for contextual factors.

Race/ethnicity	Specification	
	(a)	(b)
White	44.5% [43.6–45.3]	43.6% [42.7–44.5]
African American	54.2% [52.6–55.8]	49.4% [47.7–51.2]
Hispanic	51.3% [49.9–52.7]	48.0% [46.4–49.6]
Asian	41.0% [38.9–43.0]	44.3% [42.1–46.5]
American Indian	55.0% [50.9–59.0]	50.4% [45.5–55.3]

Note: All adjusted means are based on multivariable regression results shown in Table 2. Specification (a) adjusts for individual characteristics shown in Table 1; specification (b) adjusts for all factors, including structural influences.
